# Efficacy of adding a supporting implant in stress distribution of long-span fixed partial dentures: a 3D finite element analysis

**DOI:** 10.15171/joddd.2016.013

**Published:** 2016-06-15

**Authors:** Rami Shurbaji Mozayek, Mirza Allaf, Mohammad B. Abuharb

**Affiliations:** ^1^MSc, Department of Fixed Prosthodontics, Faculty of Dentistry, Damascus University, Damascus, Syria; ^2^Professor, Department of Fixed Prosthodontics, Faculty of Dentistry, Damascus University, Damascus, Syria; ^3^Lecturer, Department of Mechanical Design, Faculty of Electric and Mechanical Engineering, Damascus University, Damascus, Syria

**Keywords:** Finite element analysis, fixed partial denture, implant-supported dental prosthesis

## Abstract

***Background. ***Long span is seen in many clinical situations. Treatmentplanning options of these cases are difficult and may require FPD, RPD or ISP. Each option has its own disadvantages, including mechanical problems, patient comfort and cost. This article will evaluate the stress distribution of a different treatment option, which consists of adding a single sup-porting implant to the FPD by using 3D finite element analysis.

***Methods.*** Three models, each consisting of 5 units, were created as follows: 1. Tooth Pontic Pontic Pontic Tooth; 2. Tooth Pontic Implant Pontic Tooth; 3. Tooth Pontic Pontic Implant Tooth. An axial force was applied to the prostheses by using 3D finite element method and stresses were evaluated.

***Results.*** The maximum stress was found in the prostheses in all the models; the highest stress values in all the shared components of the models were almost similar. Stress in implants was lower in the second model than the third one.

***Conclusion.*** Adding a supporting implant in long-span FPD has no advantages while it has the disadvantages of complicating treatment and the complications that may occur to the implant and surrounding bone.

## Introduction

Fixed partial dentures replacing multiple missing teeth may be associated with more complications and higher failure rates.^1^ Many factors could influence the prognosis of such prostheses, including parafunction, force direction and span length. Thus, other treatment options are recommended such as implant-supported prostheses (ISP) or removable partial dentures (RPD).

There is a general agreement on the number of missing teeth that can be restored successfully; two abutment teeth can support two pontics as Tylman stated. Ante also implied that “The root surface area of the abutment teeth had to equal or surpass that of the teeth being replaced with pontics”.^2^ Another disadvantage of fabricating long-span FPD is flexing under occlusal loads, which can lead to the fracture of porcelain veneer, breakage of a connector, loosening of a retainer and an unfavorable tissue response. Flexing of FPD is related to the span length and to the cube of the length; to be more accurate, the longer the span the greater the flexing.^1^

Due to previous reasons when totally implant supported prostheses (ISP) cannot be fabricated due to anatomical limitations or any other reasons a tooth-implant-supported prostheses (TISP) could be considered.^3-5^

Tooth-implant-supported prostheses are also recommended by some authors for some selected cases.^6-10^ In TISP, use of a rigid connector is preferred over non-rigid connectors due to complications that may be seen more frequently in the latter such as tooth intrusion or peri-implantitis.^11,12^ Implant‒abutment connection type has an effect on soft tissue dimensions. Siar stated that “tapered connection tends to recapitulate soft tissue physiologic dimensions of natural tooth more than butt-joint connection”;^13^ Da Silva^14^ studied the effect of internal or external implant-abutment connection on stress distribution in TISP and concluded that external hexagon has less stress concentration and it is more preferred in TISP.

Previous studies showed that the most important factor in TISP is loading conditions^15-17^ and bone type^16^ and connector design.^14,15,18^ Other factors such as tooth-implant configuration are not yet fully understood.

In this article we discuss a mechanical solution of supporting a long-span fixed prostheses in the posterior region of the jaws with an implant as a suggested treatment option that can improve the mechanical support of poor prognosis prostheses and has lower cost compared to a fully implant-supported prostheses. Although this design was not preferred,^19^ there is still no sufficient data available to make a definitive evidence-based decision for this treatment option.

The question this study addresses is: Does adding a supporting implant to long-span FPD reduce stress?

To answer this question, virtual 3D models were designed and studied by finite element method (FEM). This method is widely used in all the fields and nothing seems to be out of reach of FEA, nuclear reactor or teeth.^20,21^

## Methods

This study did not involve the use of any animals or human data or tissues, and thus, an ethics approval was not required.

A 3D model of a long-span FPD was created by using SOLID WORKS® Premium 2011. The model represented cortical and spongy bone, teeth (dentin, cementum, pulp), periodontal ligament and a nickel‒chromium prosthesis which connected two natural teeth (as abutments) with three pontics in between.

The bone was represented as a block with a 3-mm layer of cortical bone at the neck of the teeth and the implants and a spongy bone beneath.^22^

**Figure 1:**
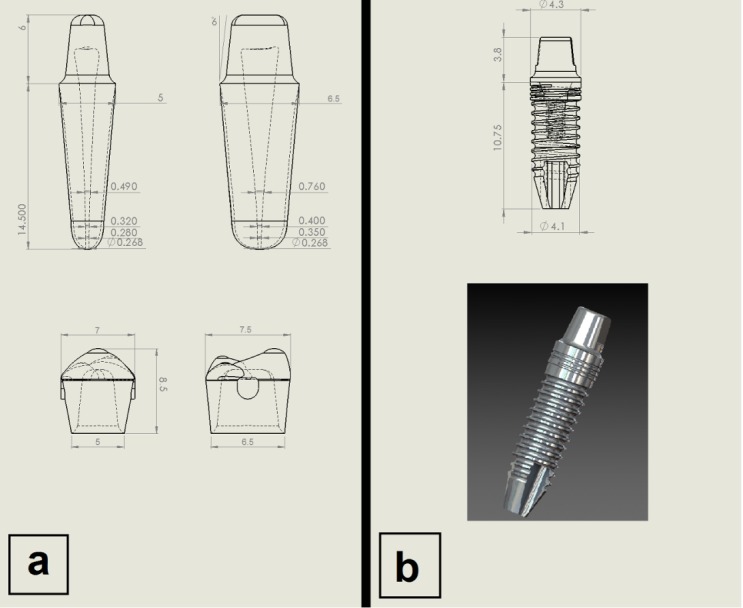
Schematic representation of the lower first premolar which was used in the study. (b) Schematic representation of NobelSpeedy™ Replace (RP 4*11.5 mm) which was used in the study.

A first lower premolar was chosen to resemble the natural teeth to be able to generalize the outcome of the study for all the teeth not only for one case with strict conditions. The premolar was constructed on average dimensions^23^ (Table 1); cementum was constructed to become gradually thicker until it reached 0.23 at the apex, while the periodontal ligament had a maximum thickness of 0.35 mm at the apex and minimum 0.1 mm at the mid-root (a mean thickness of 0.21 mm).^24^ The pulp was constructed on average dimensions, also according to the distance from the apex^25^ (Table 2). The premolars were prepared for an 0.5-mm chamfer finishing line with 6° taper (Figure 1-a). ^1^

**Table 1:** Dimensions of the modeled lower first premolar

**Buccolingual diameter of crown**	Mesiodistal **diameter of crown**	**Buccolingual diameter of crown at cervix**	**Mesiodistal diameter of crown at cervix**	**Root length**	**Crown length**
7.5 mm	7 mm	6.5 mm	5 mm	14.5 mm	8.5 mm

**Table 2:** Lower first premolar pulp dimensions in terms of the distance from the apex

**Apex size**	**Buccolingual diameter**			**Mesiodistal Diameter**		
	**1 mm from apex**	**2 mm from apex**	**5 mm from apex**	**1 mm from apex**	**2 mm from apex**	**5 mm from apex**
0.268 mm	0.35 mm	0.40 mm	0.76 mm	0.28 mm	0.32 mm	0.49 mm

The resultant model was referred to by TPPPT, T for tooth and P for pontic. In the same method, two other models were created: TPIPT and TPPIT, in which I stands for implant.

The first model (TPPPT) was used as a control model and the models TPIPT and TPPIT were used to study the effect of implant position.

NobelSpeedy™ Replace (RP 4*11.5 mm) from Nobel Biocare was selected; these dimensions were selected to be in the range of the most commonly used implants, which is 10‒12 mm for implant length^26^ and 4.1‒4.3 for implant diameter in the posterior region of the mandible or maxilla.^27^ The abutment was NobelDirect™ Posterior RP (Figure 1-b).

The prostheses were assumed perfectly fit to the tooth abutments and implant abutment with no cement layer. The objective was to eliminate the effect of cement type and thickness on stress distribution and reduce the numbers of studied variables in this study.

All the materials were assumed to be homogenous, isotropic, linear and elastic. The mechanical properties of the materials used in this study are shown in Table 3.^28,29^

**Table 3:** Mechanical properties of the materials represented in the models

**Material**	**Young’s Modulus (Pascal)**	**Poisson’s Ratio**	**Reference**
**Titanium**	1.17*10^11^	0.33	
**Dentine**	1.862*10^10^	0.31	
**Cementum**	1.8*10^ 10^	0.31	
**Pulp**	2.1*10^ 6^	0.45	Abu Nassar J
**Cortical bone**	1.37*10^10^	0.30	
**Spongy bone**	2.5*10^9^	0.30	
**Nickel-Chromium alloy**	2.05*10^ 11^	.31	
**Periodontal ligament**	5*10^7^	0.49	Rees JS

The contacts between all the bodies were assumed to be bonded in order to prevent relative motion. The models were fixed and supported from the bottom in order to allow the bone to bend under load.

An axil load was applied with a magnitude of 300 N;^30^ the loads were on the basis of ideal occlusion.^23^ For implants the load was on the center of their crowns (centrally oriented contacts).^31,32^

In all the models, finite element method with ANSYS R.13 software was used to simulate the load and calculate the stress in all of the model parts.

## Results

**Figure 2:**
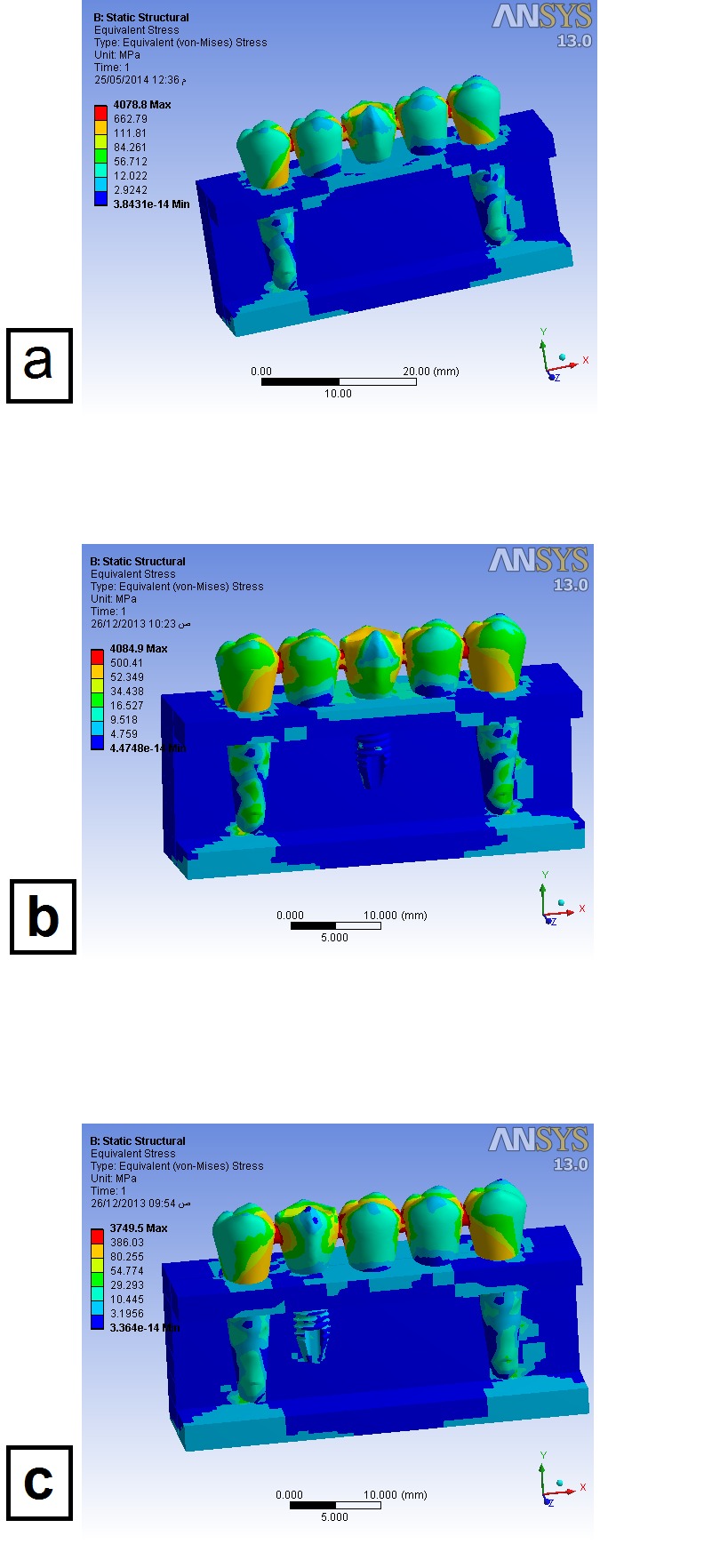
(a) Equivalent von Mises stress in the TPPPT model. (b) Equivalent von Mises stress in the TPIPT model. (c) Equivalent von Mises stress in the TIPPT model.

TPPPT: Maximum stress (equivalent von Mises stress) was located in the prostheses, especially in the connectors (Figure 2-a). Generally, stress distribution was homogeneous with some concentration in the spongy bone at the apices of teeth, whereas the finishing line at the neck of the teeth exhibited the highest stress concentration.

TPIPT: The cortical bone around the neck of the implant exhibited some stress concentration but still the highest von Mises values were found in the connectors of the prostheses (Figure 2-b). Other stress concentration locations were similar to the previous model in the spongy bone at the apices of the teeth and at chamfer finishing line.

TPPIT: This model exhibited stress distribution similar to TPIPT model with some differences in the highest von Mises values in some components of the model, primarily the implant itself (Figure 2-c).

The highest von Mises values, in each component of the models, are listed in Table 4.

**Table 4:** Maximum Von Mises in all models’ components (MPa)

**Finite element analysis model**	**Prostheses**	**Implant**	**Teeth**	**Periodontal ligament**	**Cortical bone**	**Spongy bone**
**TPPPT**	4078.8	-	144	37.891	67.83	72.051
**TPIPT**	4084.9	83.421	144.42	37.951	68.051	70.712
**TPPIT**	3749.5	231.2	142.05	38.231	42.825	70.84

In finite element analysis models, T stands for tooth, P for pontic, and I for implant.

## Discussion

Tooth‒implant connection is still controversial, with some studies advising no such connection because of the complications that may occur^33,34^like bone resorption and tooth intrusion, whereas other studies stated that TISP is an acceptable treatment option where implant-supported prostheses cannot be fabricated.^3-10^ Some authors have accepted TISP but with special conditions as rigid connectors^9,12,35^ or proper mesiodistal implant angulation.^11^ Special cases like the one presented here was not studied sufficiently and its pros and cons are still unknown; the mechanical aspects of this case are discussed here.

The finite element method is a virtual numerical analysis that can yield acceptable and reliable results if the simulation conditions are as accurate as possible. On the other hand, FEM is a subjective method that can yield different outcome if different programmers’/researchers’ visions of the loading condition, material properties and boundary conditions are taken into account. Therefore FEM cannot be a complete substitute for clinical studies but it can more likely be a guide, especially in cases that are hard to conduct or ethically not acceptable.

For this study ideal conditions were assumed like average dimensions as reported in the literature, 100% augmentation between the implant and bone, ideal occlusal contacts and average axial occlusal force.

Comparison of maximum von Mises values for all the components of TPPPT model with those of both TPIPT and TPPIT models showed no significant differences in shared components; thus no advantages in the mechanical aspect were achieved for adding a supporting implant, which might be due to the fact that periodontal ligament is the damping component and adding an implant did not increase the overall area of the ligament and consequently did not yield the desired benefit.

**Figure 3:**
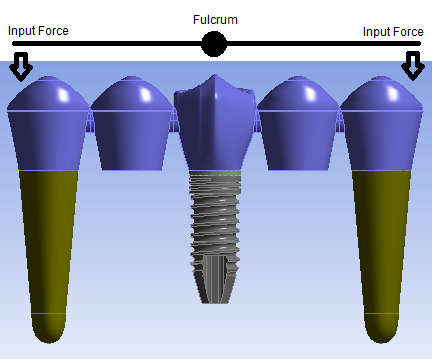
The levers in models with an intermediate implant.

Regarding von Mises values in implants, we can conclude that the position of the implant played a significant role in stress values and distributions. TPIPT exhibited lower von Mises values, which might be attributed to an increase in prostheses length whereas TPPIT had shorter prostheses in one direction but longer in the other direction. This point could be illustrated as two levers with the implant being a shared fulcrum and the teeth serving as the input force positions because of the mobility which periodontal ligaments provide (Figure 3), with longer lever arm resulting in more stress concentration around the fulcrum, i.e. the implant.

The stress in implants may lead to complications in the implant system itself such as screw loosening or even implant body fracture if significant force is applied as in bruxists. Therefore, the supporting implant had no obvious mechanical advantages but resulted in a more complicated treatment plan with more complications to worry about, consistent with previous studies^19^ that did not prefer the described design.

## Conclusions

Under the limitations of this study it can concluded that:

- The periodontal ligament plays a key role in damping loads and effectively reducing stress.

- Implant position is an important factor that the practitioner can control.

- Avoiding TISP, where possible, is a better strategy.

- Long-span with no indication for FPD is not a good candidate for TISP with implant as a supporting abutment.

## Acknowledgments

The authors acknowledge the support of Damascus University.

## Authors’ Contribution

All authors were responsible for the concept and definition of intellectual content. RSM and MA designed the study, and performed the literature search. RSM performed the experiments and drafted the manuscript. Data acquisition and analysis were performed by RSM and MBA. All authors critically revised the manuscript. All authors have read and approved the final manuscript.

## Funding

This study was supported by Damascus University.

## Competing interests

The authors declare no competing interests with regards to authorship and/or publication of this article.

## Ethics approval

Not applicable.
